# Sequence Composition Underlying Centromeric and Heterochromatic Genome Compartments of the Pacific Oyster *Crassostrea gigas*

**DOI:** 10.3390/genes11060695

**Published:** 2020-06-24

**Authors:** Monika Tunjić Cvitanić, Tanja Vojvoda Zeljko, Juan J. Pasantes, Daniel García-Souto, Tena Gržan, Evelin Despot-Slade, Miroslav Plohl, Eva Šatović

**Affiliations:** 1 Division of Molecular Biology, Ruđer Bošković Institute, Bijenička cesta 54, 10000 Zagreb, Croatia; Monika.Tunjic.Cvitanic@irb.hr (M.T.C.); Tanja.Vojvoda.Zeljko@irb.hr (T.V.Z.); Tena.Grzan@irb.hr (T.G.); Evelin.Despot-Slade@irb.hr (E.D.-S.); 2 Departamento de Bioquímica, Xenética e Inmunoloxía, Centro de Investigación Mariña (CIM), Universidade de Vigo, 36310 Vigo, Spain; pasantes@uvigo.es (J.J.P.); danielgarciasouto@gmail.com (D.G.-S.); 3 Department of Zoology, Genetics and Physical Anthropology, Universidade de Santiago de Compostela, Praza do Obradoiro, 0, 15705 Santiago de Compostela, Spain; 4 Cancer, Ageing and Somatic Mutation, Wellcome Sanger Institute, Hinxton, Cambridgeshire CB10 1SA, UK

**Keywords:** centromere, CenH3, heterochromatin, H3K9me3, chromatin immunoprecipitation, repetitive DNA, Bivalves, *Crassostrea gigas*

## Abstract

Segments of the genome enriched in repetitive sequences still present a challenge and are omitted in genome assemblies. For that reason, the exact composition of DNA sequences underlying the heterochromatic regions and the active centromeres are still unexplored for many organisms. The centromere is a crucial region of eukaryotic chromosomes responsible for the accurate segregation of genetic material. The typical landmark of centromere chromatin is the rapidly-evolving variant of the histone H3, CenH3, while DNA sequences packed in constitutive heterochromatin are associated with H3K9me3-modified histones. In the Pacific oyster *Crassostrea gigas* we identified its centromere histone variant, Cg-CenH3, that shows stage-specific distribution in gonadal cells. In order to investigate the DNA composition of genomic regions associated with the two specific chromatin types, we employed chromatin immunoprecipitation followed by high-throughput next-generation sequencing of the Cg-CenH3- and H3K9me3-associated sequences. CenH3-associated sequences were assigned to six groups of repetitive elements, while H3K9me3-associated-ones were assigned only to three. Those associated with CenH3 indicate the lack of uniformity in the chromosomal distribution of sequences building the centromeres, being also in the same time dispersed throughout the genome. The heterochromatin of *C. gigas* exhibited general paucity and limited chromosomal localization as predicted, with H3K9me3-associated sequences being predominantly constituted of DNA transposons.

## 1. Introduction

Bivalve mollusks play important roles in marine and freshwater ecosystems throughout the world, particularly in biofiltration, bioremediation, nutrient cycling and storage, stimulation of primary and secondary production, in creation and modification of habitats, biogeochemical transformations, and environmental monitoring. Their importance is highlighted when invasive bivalve species invade new ecosystems where they can achieve very high abundance, causing significant effects on the food webs [1]. Bivalves also have great significance in aquaculture, due to their high nutritional value. Their large commercial importance is reflected by a million-ton production of these organisms, as they represent a food source around the world [1]. Genome research on these organisms grows steadily. Until now, 19 bivalve genomes have been sequenced (NCBI GenBank, December 2019), the first one corresponding to the Pacific oyster *Crassostrea gigas* [2]. *C. gigas* possesses 2n = 20 chromosomes, an estimated genome size of 558 megabases (Mb), and a repeat content of 36% [2]. 

Centromeres are the chromosomal regions responsible for the accurate segregation of genetic material during mitosis and meiosis, making their proper functioning crucial for all eukaryotic organisms. Centromeres are shaped by genetic and epigenetic factors, including DNA sequences, protein components, and epigenetic marks (reviewed in [3]). One of the most prominent hallmarks of an active centromere is the presence of nucleosomes containing a specialized variant of histone H3, CenH3, known as centromere protein A (CENP-A) in mammals (reviewed in [4]). Canonical H3 histones are highly conserved in eukaryotes and differ from CenH3 histones primarily in their N-terminal tails. CenH3 proteins of different species, on the contrary, are highly divergent, being again their N-terminal ends the primary source of differences [5]. The advancement in the understanding of functional centromeres also requires elucidating which DNA sequences interact with CenH3. Centromeric regions are frequently composed of repetitive sequences, satellite DNAs (satDNAs) repeated in tandem, interspersed transposable elements (TEs), or both (reviewed in [3,6]).

To date, 26 different satDNAs have been characterized in bivalve mollusks, with Mytilidae, Ostreidae and Veneridae being the most explored families so far (reviewed in [7]). After the discovery of a Cg170 satellite DNA with 166 bp monomer-length constituting 1–4% of the *C. gigas* genome [8], subsequent cytogenetic studies have shown that these sequences are mostly centromeric [9]. Variants of the same satDNA were also found after HindIII-restriction of *C. gigas* genomic DNA, and described in more detail in several species belonging to the genera *Ostrea* and *Crassostrea* [10]. Several mobile elements have been described in detail in bivalve mollusks, belonging to retrotransposons [11,12,13] and DNA transposons [14,15,16,17]. In general, satDNAs and TEs are in many species closely linked in many different ways; reviewed in [18]. For example, some TEs have an internal region composed of a satDNA-like array of 50–500 bp long monomers that can be amplified into satDNAs [19,20]. In bivalves, *pearl* is a well-known example of element harboring short arrays of tandemly repeated monomers flanked with well-structured left and right sequence segments [14], related to ancient satDNAs found widespread in bivalve species [21]. Another structurally equivalent element, DTC84, was characterized in the clam *Donax trunculus* [16]. The presence of Cg170/HindIII repeats was also reported within DNA transposons, with varying repeat copy numbers in the internal array [22]. All those elements were recently categorized as short non-autonomous DNA transposons, able to use rolling-circle mechanisms in their propagation, and classified as members of a Helitron/Helentron superfamily of TEs [23]. Repbase database (https://www.girinst.org/repbase/, accessed on 20 September 2019) holds 87 entries belonging to such elements in the oyster *C. gigas*.

In genomes of higher eukaryotes, heterochromatin represents compacted, transcriptionally repressed chromatin, holding important functions in repetitive element silencing and genome stability maintenance. Constitutive heterochromatin is frequently defined by the presence of a specific post-translational histone modification, tri-methylation of lysine 9 on histone H3 (H3K9me3) [24]. Chromatin exhibiting heterochromatic features can be pericentromeric, intercalary or subtelomeric [25]. Available data for bivalve species indicate that heterochromatin content drastically varies among them. According to C-banding patterns, in the oyster *Ostrea angasi*, most chromosomes show centromeric C-positive regions while telomeric and interstitial C-bands are scarce [26]. Heterochromatin is abundant in *Crassostrea angulata*, occupying mostly subtelomeric and pericentric positions [27]. In the clam *Donax trunculus*, heterochromatin is mostly intercalary [28] while in *Sphaerium* species it is constricted to (peri)centromeric regions [29]. *C. gigas* exhibits very low heterochromatin content, showing a weak (peri)centromeric C-band in chromosome pair 5 and a telomeric one in chromosome pair 10 [30]. 

The annotation of the parts of the genome enriched in repetitive sequences still faces serious problems, thus, those parts are frequently missing in genome assemblies. The difficulties arise in attempts to reconstruct the exact sequential order of segments composed of highly similar repetitive sequences present in a large number of copies. Consequently, repetitive sequences are frequently omitted from datasets available in public databases, or even introduce significant errors in genome annotation [31]. For that reason, the composition of DNA sequences underlying the active centromeres and H3K9me3-marked heterochromatic regions have not been explored in the Pacific oyster, despite the existing genomic data in the form of scaffolds [2] and pseudochromosomes [32]. Therefore, in order to investigate the sequence composition of the genomic regions associated with those specific chromatin types in *C. gigas*, we employed chromatin immunoprecipitation followed by the high-throughput sequencing of the Cg-CenH3- and H3K9me3-associated DNA.

## 2. Materials and Methods 

### 2.1. Cg-CenH3 Gene Detection and Antibody Synthesis 

The bioinformatic search for the Cg-CenH3 candidate gene was performed throughout available databases using tblastn with the AA sequence of the *C. gigas* H3 protein [33] as a query. The region corresponding to the potential CenH3 candidate was found in the genome assembly “oyster.v9”, scaffold737 at the base positions: 38277–39871. The Cg-CenH3 candidate gene sequence was confirmed experimentally by PCR using the primers scaff737_38155_Fw: TGC TAC AAA CTG TCT GAC ATC G and scaff737_40133_Bw: ATA TAG GTT CGC GTT GCT TAT C. The denaturation step was performed at 94 °C for 3 min followed by 35 cycles of 94 °C for 30 s, 52 °C for 30 s and 72 °C for 30 s with a final elongation step of 72 °C for 7 min. Gene expression was confirmed by RT-PCR amplification. Total RNA was isolated from the adductor and gill tissues of *C. gigas* specimens using RNAeasy Mini Kit (Qiagen, Venlo, Netherlands). Amplification was preformed according to the protocol of One Step RT-PCR Kit (Qiagen) using the primers: CgigasCenH3_Fw1: TCA TCT GGC ACC TAA AGG AAA and CgigasCenH3_Bw1: GTC CCT TTT TCC GCT TAT TCT. The amplification conditions were: initial steps at 50 °C for 30 min and 95 °C for 15 min, followed by 34 cycles of 94 °C for 1 min, 54 °C for 30 s and 72 °C for 1 min, with final extension at 72 °C for 10 min. In order to produce a specific antibody for *C. gigas* CenH3, rabbits were immunized with a SKVTGGIKEPRNW peptide, corresponding to the N-terminal end of the Cg-CenH3 protein. Rabbit immunization and affinity-purification of the monospecific IgG-fraction was performed by Pineda Antikörper-Service Berlin, Germany. The protein sequence alignment of *C. gigas* H3 and Cg-CenH3 is presented in Figure S1a. Two proteins exhibit 56.7% similarity in their histone fold domains. Obtained Cg-CenH3 antibody was tested by western blot using protein extract derived from gill tissue of *C. gigas* (Figure S1b), preimmune serum, antiserum and antibody dilutions were 1:500.

### 2.2. DNA Barcoding

Oysters from the family Ostreidae exhibit high levels of phenotypic plasticity, which makes them a challenging group for taxonomists and phylogeneticists [34]. For that reason, DNA barcoding was performed for molecular identification and species confirmation of all bivalve specimens used in this work (2 specimens were used for the first Cg-CenH3 ChIP experiment, 9 for both H3K9me3 ChIP and the second round of Cg-CenH3 ChIP experiment, and 9 specimens were used for cytogenetic experiments). Genomic DNA was extracted using DNeasy Blood and Tissue Kit (Qiagen) from a small piece of adductor muscle (up to 25 mg of tissue) using the protocol provided by the manufacturer. The concentration of the extracted DNA was measured using Qubit Fluorometer (ThermoFisher Scientific, Waltham, MA, USA). PCR amplification was performed using primers for the mitochondrial cytochrome c oxidase subunit 1 (COI) gene, LCO-1490 5′-GGT CAA CAA ATC ATA AAG ATA TTG G-3′ and HCO-2198 5′-TAA ACT TCA GGG TGA CCA AAA AAT CA-3′ [35]. The amplification was performed with an initial denaturation at 94 °C for 5 min, 35 cycles of 94 °C for 30 s, 52 °C for 30 s, 72 °C for 30 s, and a final extension at 72 °C for 10 min. PCR products were Sanger-sequenced at Macrogen Inc. (Amsterdam, Netherlands). The sequences were compared with publicly available NCBI GenBank COI sequences of *C. gigas*.

### 2.3. Native Chromatin Isolation and Micrococcal Nuclease Digestion

Several rounds of nuclei isolation were performed from the flash-frozen adductor muscle tissue of *C. gigas*. Nuclei were isolated by homogenizing the tissue, using a Dounce homogenizer, in CIB Buffer (15 mM Tris HCl pH 7.5, 60 mM KCl, 15 mM NaCl, 0.34 M sucrose, 0.15 mM spermine, 0.5 mM spermidine) with the addition of a protease inhibitor (PI) cocktail (Roche, Basel, Switzerland), PMSF (2 mM) and Triton X-100 (0.5%). The isolated nuclei were centrifuged at 2500× *g* for 5 min at 4 °C, washed in CIB buffer without PI or detergents, and centrifugated again. The pellet was resuspended in CIB (without detergent or PI), the solution supplemented with CaCl_2_ to 1 mM and heated to 37 °C for 1 min. Micrococcal nuclease (MNase) digestion was performed using 1.5 U of MNase (Thermo scientific 88216 Lot SF251245) per mg of tissue for 5 min at 37 °C. The reaction was stopped by adding 0.5 M EDTA to a final concentration of 20 mM and samples were centrifuged at 8000× *g* for 5 min at 4 °C. The supernatant was removed and the pellet was resuspended in PBS + 500 mM NaCl and incubated at 4 °C for 2 h on a tube rotator. Afterwards, samples were centrifuged at 15,000× *g* for 15 min at 4 °C and the supernatant containing the digested chromatin collected. The extent of the degradation was checked by agarose gel electrophoresis.

### 2.4. ChIP Experiments

The mononucleosomal fraction of the MNase digested *C. gigas* chromatin was used for ChIP experiments. ChIP was performed according to the PIERCE magnetic ChIP KIT (26157) protocol with few modifications. A/G Magnetic beads were blocked in 1× PBS + 1% BSA, for 1 h at 4 °C on a tube rotator. The chromatin in ChIP buffer (0.01% SDS, 1.1% Triton X-100, 1.2 mM EDTA, 16.7 mM Tris-HCl pH 8.1, and 167 mM NaCl) plus PI was precleared on the previously blocked Dynabeads for 1 h at 4 °C on a tube rotator. Antibody binding was performed for 2 h at 4 °C in 100 µL of kit-provided “IP Dilution buffer” containing magnetic beads using anti-CenH3 or monoclonal anti-H3K9me3 (Cell Signaling 13969) antibodies. Anti-H3K4me3 (Cell Signaling 9751) and rabbit IgG (Cell Signaling 2729) antibodies were used for positive and negative controls, respectively. All antibodies were added in the amounts suggested by the manufacturers (1 µL per µg of chromatin). An amount of 4.6 µg of *C. gigas* chromatin was used for each of the ChIP reactions. ChIP using anti-Cg-CenH3 was performed in two subsequent biological replicates, and anti-H3K9me3 in one. The beads with bound antibody were mixed with the precleared chromatin and the mixture incubated overnight at 4 °C on a tube rotator. The immunoprecipitated material was washed, eluted and further processed according to the PIERCE magnetic ChIP KIT protocol.

### 2.5. Sequencing, Read Clustering, and ChIP-Seq Data Analysis

Next-generation sequencing (NGS) library preparation and sequencing of one genomic DNA sample, one Input, and one ChIP sample (the first biological replicate using anti-Cg-CenH3) were performed by Admera Health facility (USA) on the Illumina HiSeqX platform, using multiplexing of the samples in a single lane. One anti-H3K9me3 ChIP sample and the second biological replicate of Cg-CenH3 ChIP sample, together with their corresponding Input, were sequenced later on using the same platform, again with sample multiplexing. Low-coverage sequencing was employed for all NGS data, as for repetitive DNA analysis significantly reduced genome coverage has been recommended (0.01–0.50×) [36]. When genome sequencing data of low-coverage are used, reads that originate from repetitive elements produce multiple similarity hits. They can thus be identified as clusters of frequently overlapping sequences, while almost no similarity is detected between reads derived from single-copy sequences. The number of reads in each cluster is proportional to the genomic abundance of the corresponding repeat, making this the strategy of choice in repetitive sequence detection [36]. In order to obtain a suitable reference needed for the identification of Cg-CenH3 and H3K9me3 ChIP-enriched sequences, the genome of the Pacific oyster was sequenced at about 1.5× coverage, obtaining pair-end reads (2 × 2,768,912 reads, 151 bp in length). The first round of sequencing produced 2 × 2,419,817 reads for Cg-CenH3 ChIP and 2 × 2,518,230 for the Input sample, all 151 bp in length. The second round of sequencing generated 2 × 5,570,873 reads for the Input sample, 2 × 6,463,282 for Cg-CenH3 ChIP, and 2 × 5,516,696 for H3K9me3 ChIP, all 151 bp in length. Raw sequence reads from WGS and all ChIP and Input samples can be found under the BioSample accessions: SAMN15184427, SAMN15184428, SAMN15184429, SAMN15184431, SAMN15184432, SAMN15184433, NCBI BioProject ID: PRJNA638244. 

Genomic repeat identification, which is a prerequisite for ChIP-Seq Mapper analysis, was performed by similarity-based clustering of reads in the RepeatExplorer2 pipeline [37] of the Galaxy server (https://repeatexplorer-elixir.cerit-sc.cz/galaxy/), using default parameters (accessed June 2019). For that purpose, one million genomic reads (quality filtered, trimmed, interlaced, without overlap) were randomly subsampled from NGS genomic dataset and used for clustering. After clustering, each contig was treated separately in the ChIP-Seq Mapper analysis to obtain more distinct enrichments, as in certain cases clusters contain multiple contigs of limited similarity. Quality filtering, trimming, and subsampling of one million reads was also performed for ChIP and Input datasets. Cg-CenH3 and H3K9me3 ChIP-Seq reads and reads from the Input samples were mapped to contigs via the ChIP-Seq Mapper tool implemented within Galaxy server. The proportion of ChIP and Input reads mapped to certain contig was inspected to identify repeats with ChIP/Input ratio > 1.7 and number of hits in ChIP sample > 500, which were considered to represent sequences enriched in the ChIP sample. For Cg-CenH3 ChIP reactions, sequences that were present in both biological replicates and met the aforementioned criteria were considered as centromeric candidates. Selected criteria were approximating enrichments used in studies where CenH3 was shown to be associated with many different sequences, e.g., [38,39].

### 2.6. Mitotic Chromosomes and Gonadal Suspension Preparations

Juvenile oyster specimens were collected from their natural habitat at Aveiro, Ria de Aveiro, Portugal. Oysters were kept in the laboratory in tanks with aerated, filtered seawater at 18 ± 1 °C and fed on microalgae for seven days to promote somatic and gonadal growth and maturation. The species was determined according to shell characteristics and further confirmed by PCR using primers for COI gene, as previously described. The mitotic chromosome preparations were obtained as described in Martinez-Exposito et al. [40], with slight modifications. After 12-h treatment of the specimens in 0.005% colchicine solution, gills and gonads were excised. Gills underwent a hypotonic shock in 50% and 25% seawater (25 min each) and then were fixated in ethanol:acetic acid (3:1) for 1 h. The cell suspension obtained by treating dissected gills in 60% acetic acid was dropped onto slides previously heated to 56 °C. The slides were air-dried.

After isolating gonads, gonadal suspensions were inspected under a ZEISS SteREO Discovery.V20 microscope for sex determination. Gonadal cell suspensions from male specimens were centrifuged for 30 s at maximum speed using a benchtop microcentrifuge. Gonadal cells were then fixed with 3.7% paraformaldehyde with 5% acetic acid and 0.9% NaCl or methanol:acetone (1:1), and dropped onto poly-*D*-Lys pretreated slides (methanol:acetone yielding cleaner preparations). After covering with a coverslip and squashing, the slides were briefly submerged into liquid nitrogen, the coverslips removed, and the slides stored at −80 °C. Maturation stages of gonadal cells were determined according to [41].

### 2.7. Probe Labelling

Probes for fluorescent in situ hybridization (FISH) of sequences belonging to Cg170, Cl112, Cl150, Cl344, Cl460 and Cl485 were PCR-labelled in 50 µL volumes, containing 250 ng of DNA, 50 µM dATP, dGTP and dCTP, 32 µM dTTP and 18 µM biotin-16-dUTP, primers (1 µM each), 2.5 mM MgCl2, 2.5 U GoTaq G2 Flexi Taq DNA polymerase and 1× GoTaq Flexi Reaction Buffer (all Promega). The initial denaturation was performed at 94 °C for 5 min and was followed by 30 cycles of amplification under the conditions shown in Table S1 for each set of primers. The final extension was done at 72 °C for 7 min. Probes were purified using QIAquick PCR Purification Kit (Qiagen) according to the manufacturer’s protocol. Probes were checked on 1% agarose gel for quality and the concentration of the purified probes was measured using a Qubit Fluorometer. Probes for the detection of CenH3- and H3K9me3-associated sequences were biotin-labelled by the random priming method, using 66 ng and 48 ng of ChIP-ed DNA, respectively. 

### 2.8. Fluorescent In Situ Hybridization (FISH)

FISH experiments were performed according to the protocol described in Pérez-García et al. [42] with the adaptation in pepsin digestion (5 min at 37 °C). Prior to application, probes were denatured at 80 °C for 8 min and cooled on ice for 2 min. Detection was performed using fluorescein-labelled avidin D (Vectashield) and biotinylated anti-avidin D (Vectashield). Slides were counterstained with 4′, 6-diamidino-2-phenylindole (DAPI; 100 ng/mL) and mounted with Mowiol 4-88 antifade mounting medium (Sigma-Aldrich, St. Louis, MO, USA). Slide visualization was performed using a Nikon Eclipse-800 fluorescent microscope and a Leica TCS SP8 X laser scanning microscope.

### 2.9. Immunofluorescence (IF)

Several attempts of protein visualization were employed on mitotic chromosomes prepared as described, using custom made Cg-CenH3 antibody (dilutions 1:200 and 1:400) as well as two commercially available antibodies against H3K9me3 (Cell Signaling 13969 and Merck 05-1242) diluted 1:800 and 1:75, respectively, and H3 (Cell Signaling 4499), diluted 1:400. However, protein localization on cytological material prepared from this species in this way was disabled due to high concentration of acetic acid (a part of fixative necessary for obtaining metaphase chromosomes), destroying protein epitopes.

Several other fixatives have been tried on both gonadal and gill tissue: methanol:acetone = 1:1; 3.7% paraformaldehyde with 5% acetic acid and 0.9% NaCl; ethanol:acetic acid (3:1); and methanol:acetic acid (3:1). We also examined two different ways of the slide preparation: air-drying the slides after dropping the cell suspension, and squashing the slides after dropping the cell suspension. All tested conditions resulted in no IF-utilizable material (too much unresolved tissue remained on the slides and/or metaphase chromosomes could not be obtained).

Therefore, we performed localization and colocalization experiments on interphase nuclei of male gonadal cells in different phases of maturation, fixed without highly concentrated acetic acid, and prepared as already described in “gonadal suspension preparations”. In continuation, slides were washed in 1× PBS for 2 min and two times in 1× PBS/0.2% Tween 20 for 5 min. Blocking was performed with 2.5% BSA in 1× PBS/0.2% Tween 20 at 37 °C for 1 h. Afterwards, the slides were incubated with rabbit anti-CenH3 primary antibody, diluted 1:400 in 1× PBS containing 0.2% Tween 20 and 2.5% BSA, overnight at 37 °C in a humid chamber. In continuation, slides were washed three times in 1× PBS/0.2% Tween 20. Washing was followed by 1 h incubation with a secondary antibody (goat anti-rabbit IgG labeled with Alexa 594 fluorophore, Abcam ab150080) diluted 1:1000 in 2.5% BSA in 1× PBS/0.2% Tween 20, at 37 °C in a humid chamber. Subsequent washes were performed two times in 1× PBS/0.2% Tween 20 for 5 min and one time in 1× PBS for 2 min. Slides were counterstained with DAPI and mounted with Mowiol 4–88 mounting medium.

Double immunofluorescence experiment was done according to the same protocol. The primary antibodies used were custom-made rabbit anti-Cg-CenH3 antibody (Pineda Antikörper-Service), diluted 1:400, and mouse anti-H3K9me3 antibody (Merck 05-1242), diluted 1:75. Secondary antibodies used were goat anti-rabbit IgG labeled with Alexa 594 fluorophore (Abcam ab150080, 1:1000), and donkey anti-mouse IgG labeled with Alexa 488 fluorophore (Sigma-Aldrich, SAB4600035, 1:500). Slides were incubated simultaneously with both types of primary antibodies in the first incubation, and with both types of secondary antibodies in the second incubation.

### 2.10. IF-FISH

IF-FISH experiments were performed according to the protocols described previously for each method. After the incubation with the secondary antibody and subsequent washes with PBS/Tween and PBS in the IF experiment, the slides were not counterstained with DAPI. Instead, the FISH experiment followed. RNAse treatment was performed for 45 min and the pepsin treatment was omitted. The rest of the experiment was performed as already stated.

### 2.11. Localization of Cg170 on Pseudochromosomes

The pseudochromosomes of *C. gigas* (ftp://ftp.ifremer.fr/ifremer/dataref/bioinfo/) are from a pseudochromosome assembly of the Pacific oyster genome, generated using a combination of BAC-end sequencing and scaffold anchoring to a new high-density linkage map [32]. They represent, however, only 50.1% of the total length of the oyster_v9 assembly [1] (http://www.oysterdb.com), omitting (longer) segments composed predominantly of repetitive sequences. Due to the mentioned limitations, pseudochromosomes were used only for an informative overview of the general distribution of Cg170 sequences across the the *C. gigas* chromosomes. The annotation of the sequences of interest on the pseudochromosomes of *C. gigas* was performed locally using Geneious 9.1.7 (Biomatters Ltd., Auckland, New Zealand). 

## 3. Results

### 3.1. DNA Repeat Content of the C. gigas Genome 

In order to obtain a reference database for the analysis of the composition of the ChIP-obtained sequences, RepeatExplorer clustering was performed on paired-end NGS short reads derived from *C. gigas* genomic DNA. For that purpose, a subset containing one million NGS reads was randomly sampled from the pool of genomic sequences. Sequence clustering resulted in 83,856 clusters, and those constituting ≥ 0.01% of the genome were classified by the RepeatExplorer pipeline (Table 1). According to the performed analysis, satDNAs and mobile elements turned out to be similarly abundant repeat groups, with relatively low genome contribution, below 3% for each group. Among mobile elements, Ty3-gypsy retrotransposons were found to occupy 0.09%, and LINE retrotransposons 0.11% of the genome. The most abundant satellite DNA was Cg170, constituting 1.76% of the genome, and 68.75% of all of the detected satellite DNA repeats. 

After the genomic DNA clustering, CenH3 and H3K9me3 ChIP-obtained reads and reads from the Input samples were mapped to clustering-obtained contigs via ChIP-Seq Mapper tool. 

### 3.2. Centromeric Sequences of C. gigas 

**Figure 1:**
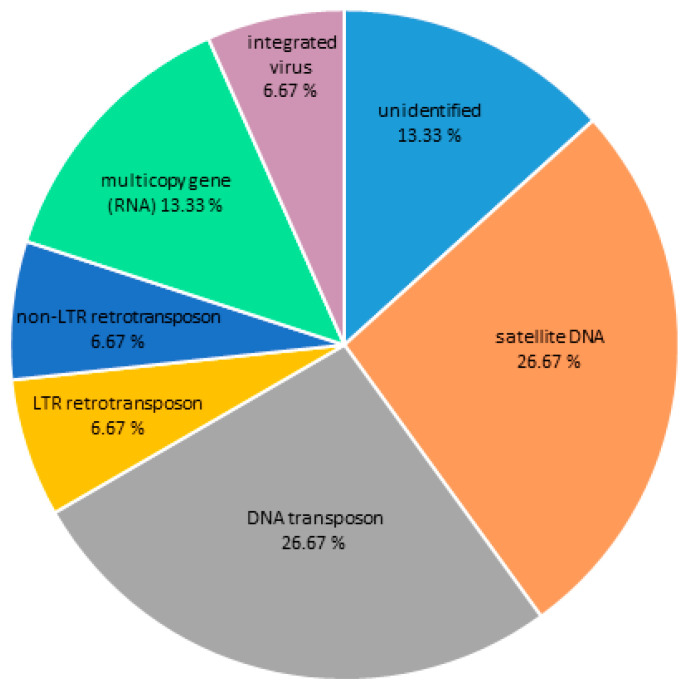
The DNA sequence composition of the Cg-CenH3-associated chromatin of *C. gigas* as assigned to main repeat groups based on Repbase and RepeatExplorer classification.

Contigs with ChIP/Input ratio > 1.7 and number of hits in ChIP sample > 500 for two Cg-CenH3 ChIP reactions were considered to represent centromeric candidate sequences of *C. gigas*. Sequences enriched in the Cg-CenH3 ChIP samples and their characteristics are presented in Table S2. The main repeat groups found in association with CenH3 are DNA transposons (26.67%), ribosomal genes (13.33%), LTR retrotransposons (6.67%), non-LTR retrotransposons (6.67%), integrated virus (6.67%) and satDNA (26.67%), while 13.33% belonged to non-identified sequences (Figure 1). In Table S2, inability of Repbase and RepeatExplorer to unambiguously classify the repeat was marked as “other” or “N/A”. 

**Figure 2:**
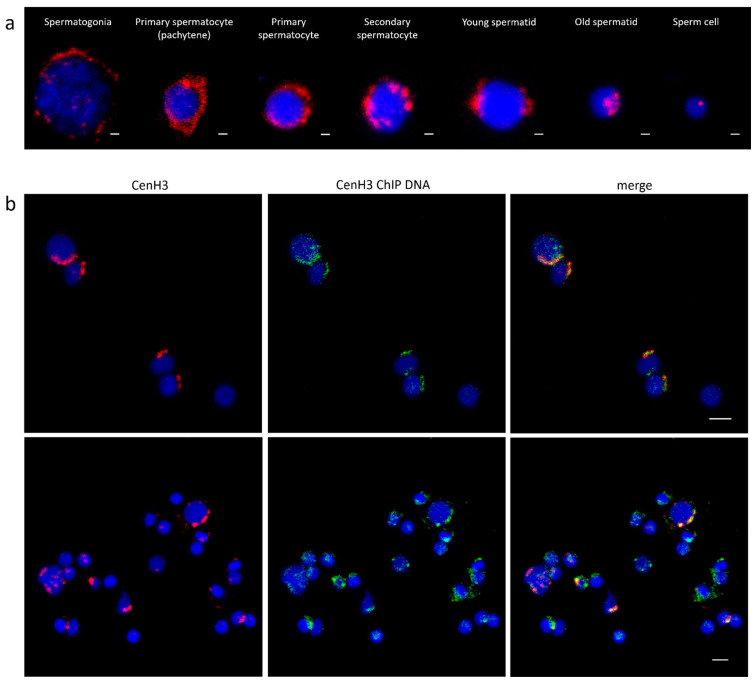
Location of Cg-CenH3 and centromeric DNA in *C. gigas*. (**a**) Distribution of Cg-CenH3 in male *C. gigas* gonadal cells in different stages of maturation. Scale bar = 1 µm. (**b**) Colocalization of Cg-CenH3 protein and complete Cg-CenH3-immunoprecipitated DNA on gonadal cells. Scale bar = 3 µm.

The cytological colocalization of all immunoprecipitated sequences with Cg-CenH3 was assessed using fluorescence in situ hybridization (FISH) combined with protein immunofluorescence (IF) detection. However, in attempts to localize Cg-CenH3 protein on chromosomes, we found that acetic acid (a component of the fixative necessary for obtaining metaphase chromosomes) most likely destroys epitopes, disabling the protein localization on this type of material. For that reason, colocalization was performed on interphase nuclei of male gonadal cells in different phases of maturation. The distribution patterns of Cg-CenH3 signals differ along the stages of maturation of the gonadal cells (Figure 2a). IF-FISH using an anti-Cg-CenH3 antibody and complete Cg-CenH3-immunoprecipitated labelled DNA is presented in Figure 2b. It is noticeable that immunostaining signals using the anti-Cg-CenH3 on interphase nuclei overlap with a substantial part of the FISH signals derived from complete ChIP-obtained DNA sequences, although, due to the reasons discussed in the following sections, the overlap is not absolute. 

**Figure 3:**
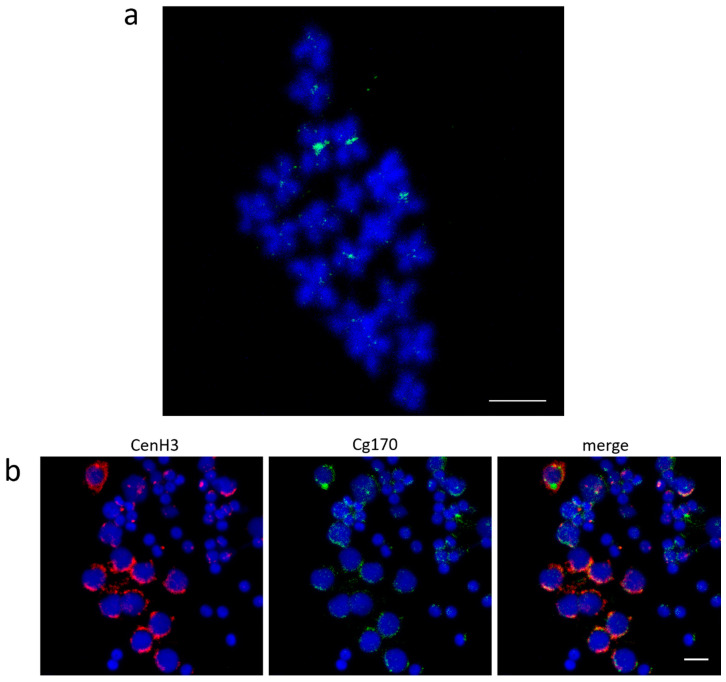
Localization of Cg170 repeats and Cg-CenH3 in *C. gigas*. (**a**) Mapping of Cg170 repeats to metaphase chromosomes. (**b**) Colocalization of Cg-CenH3 protein and Cg170 repeats on gonadal cells. Scale bars = 5 µm.

The association of Cg-CenH3 signals with centromeric regions was also indirectly confirmed using FISH with Cg170 probe combined with immunodetection of Cg-CenH3 (Figure 3). Cg170 satellite DNA is known to occupy primary constrictions of multiple chromosomes of *C. gigas* [9], but is not present in Table S2 as it did not satisfy the enrichment criteria for both ChIP reactions. Due to sub-variants of the Cg170 sequence, this repeat was found to be present in several clusters after the initial clustering of the genomic DNA (Cl1 Cl2, Cl3, Cl41, and Cl60). Subsequently, its ChIP/Input ratio in the ChIP-Seq Mapper analysis was 1.7 in ChIP 1 and 1.2 in ChIP 2 for Cl1, and the ChIP/Input ratio was 1.3 in both ChIP experiments for Cl3. The rest of the Cg170-containing clusters exhibited enrichment from 0.6–1.5 if the number of hits in the ChIP sample was >500 or the ChIP/Input value was up to 5, but in that case, the number of hits in the ChIP sample was as low as ≤10. 

FISH experiments on metaphase chromosomes using the Cg170 probe display signals in centromeric regions of several chromosomes of *C. gigas*, together with interspersed signals on chromosome arms (Figure 3a). A comparable distribution pattern is noticeable on male gonadal cells (Figure 3b). There, Cg-CenH3 signals overlap with a large part of the Cg170 FISH signal, but part of the FISH signal remains interspersed and devoid of CenH3. High interspersion of the Cg170 sequence along chromosomes of the Pacific oyster was also confirmed by annotation of this sequence in silico on pseudochromosomes of *C. gigas* (Figure S2). Such distribution could be connected with the fact that Cg170 tandem repeats constitute a central part of the Helitron-N55_CGi, as well as of several other elements of the same family (Table S3). It should be also stated that the CL16_69 sequence, found enriched in Cg-CenH3-ChIP (Table S2), belongs to the sequence segment neighboring the Cg170 central repeats of Helitron-N55_CGi.

**Figure 4:**
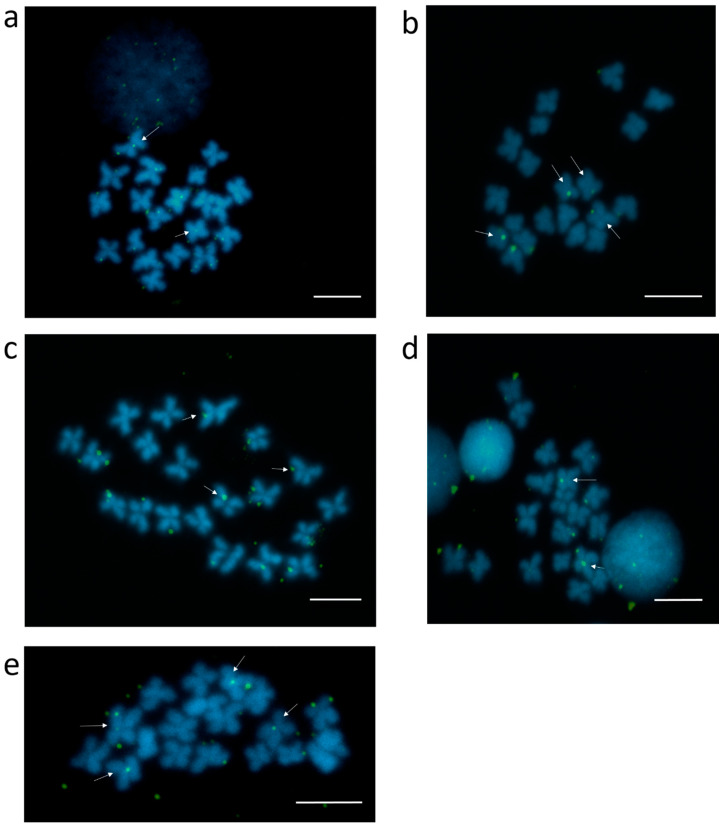
Localization of sequences belonging to several clusters enriched in Cg-CenH3 ChIP on metaphase chromosomes of *C. gigas*. Cl112 (**a**), Cl150 (**b**), Cl344 (**c**), Cl460 (**d**) and Cl485 (**e**) Signals located at the centromeres are marked by arrows. Scale bars = 5 µm.

It has to be taken into consideration that a large part of repetitive sequences enriched in Cg-CenH3 ChIP belong to different classes of interspersed sequences, potentially also residing outside the centromeric regions of *C. gigas* chromosomes. Several candidates from Table S2 were selected for mapping on metaphase chromosomes of *C. gigas*, taking into account their enrichment and/or classification, with preference to sequences repeated in tandem (satDNAs and Helitrons harboring central repeats), due to higher probability of their visualization. For that reason, sequences belonging to Cl112, Cl150, Cl344, Cl460, and Cl485 were selected to be further processed. Clusters Cl282, Cl455 and Cl460, due to ~80% sequence similarity among them, were treated as one group. FISH using probes for clusters Cl112, Cl150, Cl344, Cl460 and Cl485 displayed centromeric localization on 1–2 chromosome pairs for all inspected sequences (Figure 4), together with an interspersed pattern along chromosome arms. 

**Figure 5:**
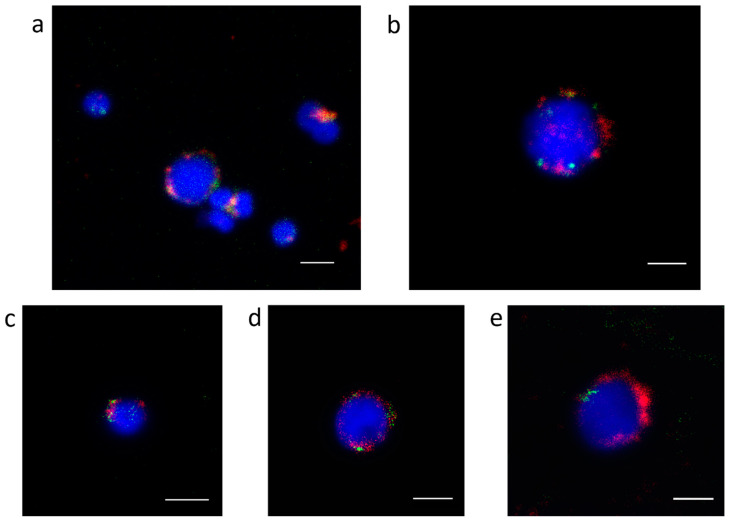
Colocalization of Cg-CenH3 protein (**red**) and sequences belonging to several clusters enriched in Cg-CenH3 ChIP (**green**) on gonadal cells. Cl112 (**a**), Cl150 (**b**), Cl344 (**c**), Cl460 (**d**) and Cl485 (**e**). Scale bars = 3 µm.

IF-FISH colocalization on gonadal cells was performed for several centromeric candidates, again displaying an overlap of the Cg-CenH3 signals with part of the FISH signals derived from sequences belonging to Cl112, Cl150, Cl344, Cl460 and Cl485 (Figure 5). 

### 3.3. Heterochromatic Sequences of C. gigas 

**Figure 6:**
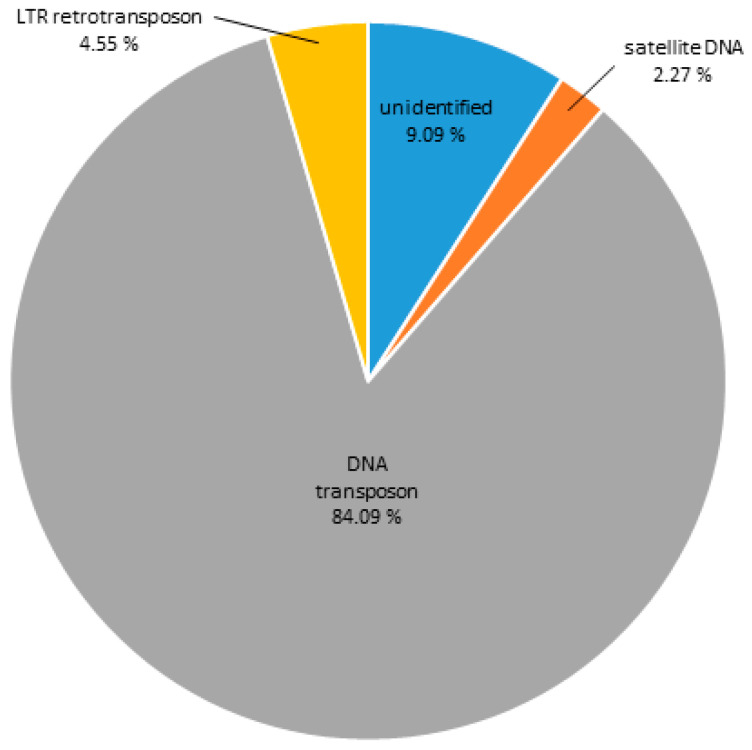
DNA sequence composition of H3K9me3-associated chromatin of *C. gigas* as assigned to main repeat groups based on RepBase and RepeatExplorer classification.

In order to address the analysis of the DNA sequences constituting H3K9me3-hetrochromatin, the same criteria were applied when determining ChIP enrichment (ChIP/Input > 1.7; number of hits > 500 in the ChIP sample). Contigs enriched in the H3K9me3 ChIP sample and their classifications are presented in Table S4. The main repeat groups found in association with this heterochromatic mark are shown in Figure 6. DNA transposons are the most abundant repeats in the H3K9me3-ChIP sample, comprising 84.09%, while satDNAs constitute only 2.27%. LTR retrotransposons build up 4.55%, and 9% of the reads were classified as unidentified repeats.

**Figure 7:**
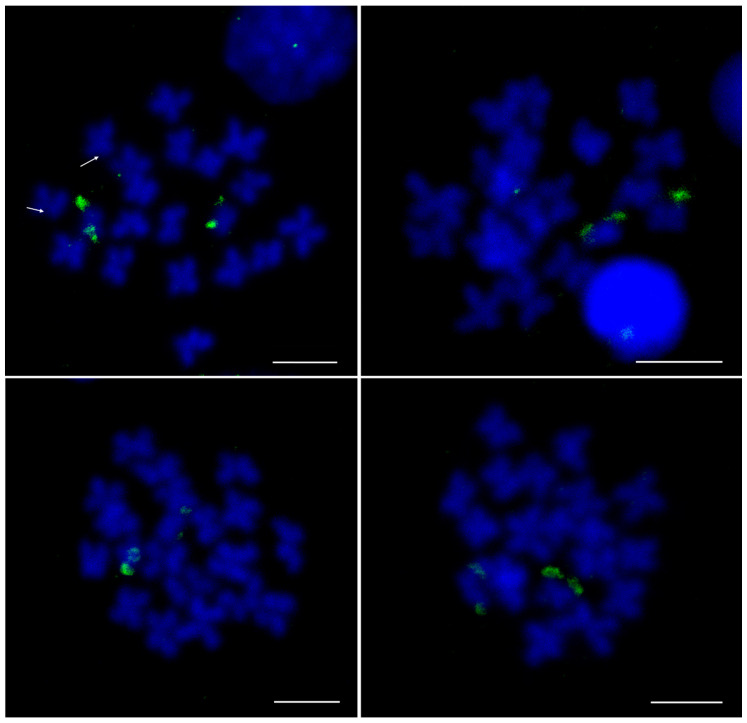
FISH localization of H3K9me3-immunoprecipitated DNA on metaphase chromosomes of *C. gigas*. Note the strong telomeric signal on the smallest chromosome pairs. Arrows in the upper left panel indicate weak centromeric signals. Scale bars = 5 µm.

The FISH experiment on metaphase chromosomes with the H3K9me3-ChIP DNA probe exhibited strong signals on telomeric regions of the smallest chromosome pair and weak signals in the (peri)centromeric region of another pair (Figure 7). In addition, the relative positioning of two epigenetic marks, Cg-CenH3 and H3K9me3, was explored. For that purpose, double IF experiments were performed on male gonadal cells (Figure S3). Immunostaining using anti-H3K9me3 revealed signals showing only limited overlap with Cg-CenH3 signals. Two strong H3K9me3 signals reside outside of the Cg-CenH3-marked centromeric area, while weak H3K9me3 signals overlap with the Cg-CenH3, in agreement with the result observed in Figure 7.

## 4. Discussion

In this work, repeat content of *C. gigas* genome was accessed via RepeatExplorer clustering (Table 1). It can be noticed that, by employing this pipeline, and inspecting clusters that represent >0.01% of the genome, a large amount of repetitive sequences (~75%) remained unidentified/unclassified. A similar problem was reported by Zhang et al. [2], who performed homology searches and ab initio prediction of repetitive sequences in *C. gigas* genome and revealed that >62% of the detected repeats could not be assigned to known categories. Difficulties in the analyses of repetitive sequences in *C. gigas* were also observed in other bivalve species. Ab initio prediction of repetitive elements in several bivalve genomes (*M. galloprovincialis*, *C. gigas*, *L. gigantea*, *P. fucata*, *A. californica*), showing repeat contents between 22.47 and 43.70%, consistently demonstrated that large parts (>70%) of the detected repeats remained unclassified for almost all bivalve species inspected [43]. One of the reasons that could explain this poor classification of repetitive sequences in bivalves is the high contribution of “combined” repetitive sequences, i.e., mobile elements that contain tandem repeats in their structure. These are mobile elements of the Helitron superfamily that usually hold arrays of tandem repeats in their central part, flanked with left and right sequence segments [23]. Although the RepeatExplorer analysis performed in this work did not report any Helitron element, 87 entries of such elements can be found in Repbase database for *C. gigas*. In this sense, several clusters showing similarity to Repbase Helitrons (Tables S2 and S4), were classified differently within the RepeatExporer cluster report. Cl16 and Cl112 (Table S2) and Cl18, Cl20, Cl24, Cl29, Cl42, Cl105, Cl149 and Cl188 (Table S4) remained unclassified (stated as “other”). In some cases, tandem repeats from central parts of sequences recognized as Helitrons in Repbase were placed in one cluster, and classified as satellite DNA, while sequences surrounding central repeats were assigned to other clusters, without clear classification. One such example is a Helitron (Table S3), whose central repeats, composed of monomers of Cg170 satDNA [8,22], are clustered separately from their left and right flanking sequences, that were placed in Cl14 and Cl16. Using the same pipeline, Silva et al. [44] describe similar classification problems in *Drosophila*. They observed that the 145TR repeat, known to be part of a Helitron element [45], was classified as a low confidence satellite. The same classification was attributed to the 225TR repeat, previously identified as a component of the intergenic spacers of the ribosomal genes [45], to the pvB370 repeat, derived from the terminal direct repeats of the pDv transposable element [46], and to the 36TR tandem repeat found within pDv element [46].

The most abundant tandem repeats in *C. gigas* genome belong to Cg170 family, building 1.76% of the genome, and 68.75% of estimated total content of tandem repeats (Table 1). Our results are concordant with previous estimates of this satDNA composing 1–4% of the *C. gigas* genome [8]. Although Cg170 repeats did not satisfy the criteria of ChIP-enrichment (ChIP/Input > 1.7; >500 hits) in both ChIP reactions, their contribution to the constitution of the centromere cannot be completely ruled out. First, due to their centromeric cytological localization on 4-7 out of 10 chromosome pairs, reported previously [9] and confirmed in this work (Figure 3a), and, second, because of their colocalization with Cg-CenH3 on male gonadal cells of *C. gigas* (Figure 3b). The important feature of Cg170 repeats appear also to be its distribution along the whole genome, as observed on male gonadal cells, on chromosomes by FISH, and *in silico* on pseudochromosomes. Besides, although about 50% of the genome are missing on pseudochromosomes, annotation (Figure S2) shows large amounts of Cg170 short arrays along chromosome arms, most of them being too short to be detectable by FISH on metaphase chromosomes (Figure 3a). This interspersion pattern (Figure S2) is most probably a direct result of Cg170 embedment into Helitron mobile elements (Table S3), as anticipated in Repbase and by our previous observations [22]. Therefore, it could be speculated that these sequences do not exhibit noteworthy ChIP/Input enrichment due to their significant interspersion and abundance along the chromosomes of *C. gigas*, in addition to their presence at centromeres. However, the enrichment of Cl16 sequences in the Cg-CenH3 ChIP sample (Table S2) would indicate that Cg170 could also be present at centromeres inside Helitrons, as Cl16 are composed of sequence segments that follow Cg170 central repeats within such elements. High numbers of tandemly repeated Helitrons at centromeres are not unusual, and were previously reported for 27 plant species [47]. These Helitrons were often truncated, as noticed for CentHels from rice and maize. *Arabidopsis thaliana* also harbored hundreds of tandemly repeated truncated Helitrons in five centromeres, interspersed with some genes. A similar situation was also observed in *Solanum tuberosum* and *S. lycopersicum* genomes, where arrays of truncated Helitrons residing in centromeres are interspersed with genes and other types of repeats [48,49]. As both Cl14 and Cl16 sequences surround Cg170 repeats in Helitrons, the presence of only Cl16 among the enriched sequences in Cg-CenH3 ChIP (Table S2) could imply that a truncated form of this element can be potentially found at *C. gigas* centromeres. Such truncations of one side of the Helitron elements are a frequent result of the rolling-circle replication (RCR) process employed by these elements, where partial RCR can amplify sequences at Helitron 5′-ends more times than those at 3′-ends [47].

DNA sequences in clusters Cl33, Cl43 and Cl69 (Table S2) show similarity to ribosomal genes. The nuclear genes for ribosomal RNA in eukaryotes are organized in two multigene families: major (45S) rDNA composed of genes coding for 18S, 5.8S and 28S rRNAs separated by two transcribed spacers, and minor (5S) rDNA repeats which code for the 5S rRNA and a non-transcribed spacer [50]. Tandem repeats of rDNA form clusters at different chromosomal locations, constituting the nucleolar organizing regions (NORs) [40]. rDNA sequence derivatives can be also found as tandem repeats showing homology to intergenic sequences [51,52,53,54]. Chromosomal mapping revealed the centromere-proximal location for some of rDNA clusters in different bivalve species [55,56,57,58,59], as well as in other organisms, such as in the plant *Arabidopsis thaliana* [60]. In addition, Robicheau et al. [61] report rDNA sequences appearing at multiple sites on chromosomes without forming rDNA arrays, presence of centromere-associated rDNA hits, and divergence of these sequences to pseudogenes. Yang et al. [62] even demonstrated that a 5S ribosomal RNA gene array was recruited to be the functional centromere for one of the switchgrass chromosomes, making Pv156 repeat the most enriched centromeric repeat. Thereafter, association of rDNA sequences with the centromeric region of *C. gigas* (Table S2) is not unexpected. 

In our IF experiments using Cg-CenH3 antibodies, we revealed the stage-specific localization pattern of this protein on male gonadal cells (Figure 2a), but due to the technical constraints failed to show its localization on metaphase chromosomes. Although chromosomal territories have not been described in *C. gigas*, it is evident that the distribution of the Cg-CenH3 signals on male gonadal cells is reproducible and non-random. A similar distribution pattern has been observed for centromeric DNA sequences across different stages of mouse and human gonadal cells [63], and correlated with the distribution exhibited by the RBMY protein [64], a paralogue of RBMX, which is in turn a component of the centromere complex involved in cohesion regulation [65,66]. Centromere localization on sperm cells corresponding to one observed in *C. gigas* in this work (Figure 2a), was noticed also in humans [67], and in grasshoppers [68]. It could therefore be concluded that despite the inability to detect Cg-CenH3 on metaphase chromosomes, its distribution on gonadal cells of *C. gigas* and combined colocalization experiments are trustworthy. 

Our colocalization experiments on interphase nuclei (Figure 2b) show overlapping of the anti- Cg-CenH3 immunostaining signals with a large part of the FISH signals of the complete ChIP-obtained DNA probe. The rest of FISH signals, residing outside of the Cg-CenH3 area, are very likely due to interspersed nature of sequences that are building the centromere, and their presence also along the chromosome arms. Consistently, many of the enriched sequences were assigned either to mobile elements (Table S2) or to unclassified sequences, “root-related repeats”, exhibiting satellite DNA characteristics, again, likely associated with mobile elements. The latter most probably belong to yet undescribed Helitron mobile elements harboring tandem repeats. 

Chromosomal mapping of H3K9me3-associated sequences on metaphase chromosomes (Figure 7) revealed strong signals at the telomeric region of one chromosome pair and weak signals on the (peri)centromere of another pair. Such distribution is consistent with C-banding results for *C. gigas* obtained by Bouilly et al. [30]. They reported very low heterochromatin content in this species consisting of a weak (peri)centromeric C-band on chromosome pair 5, and of a strong telomeric C-band on chromosome pair 10. The scarcity of H3K9me3-marked heterochromatin, showing very little overlapping with the Cg-CenH3 signal, was also confirmed by double IF experiments performed on gonadal cells (Figure S3). As most heterochromatin in *C. gigas* is telomeric (Figure 7, [28]), it is not surprising that the signal distribution pattern along *C. gigas* spermatogenesis closely resembles the distribution pattern of telomeric sequences along mice male gonadal cells [63,69]. 

Comparing the Cg-CenH3- and H3K9me3-immunoprecipitated sequences, repeats showing enrichment were more diverse in Cg-CenH3 chromatin (Figure 1 and Figure 6). Contigs enriched in ChIP-Seq Mapper analysis for Cg-CenH3-associated sequences belong to 15 genomic clusters and six repeat types. H3K9me3 ChIP enriched contigs belong to 49 clusters and three repeat types, showing strong predominance of DNA transposons. 

As already noted, some of the Cg-CenH3- and H3K9me3-immunoprecipitated sequences remained unclassified (Tables S2 and S4). CenH3-associated sequences were also partially unclassified in other species, e.g., *Saccharum spontaneum* [39] and *Beta vulgaris* [38]. Although in *C. gigas* Cl112, Cl150, Cl344, Cl460 and Cl485 sequences are Cg-CenH3-associated, and displayed centromeric localization, they appeared on only one or two chromosome pairs (Figure 4). Even the most abundant satDNA, Cg170, does not encompass centromeres of all chromosomes of the Pacific oyster (Figure 3a). The diversity of sequences present in the Cg-CenH3-associated chromatin (Table S2), together with the chromosomal distribution of a subset of selected sequences (Figure 3a, Figure 4), indicate that the chromosomes of *C. gigas* do not show uniformity in the DNA sequences constituting their centromeres. In some organisms, CenH3-associated sequences show very little diversity, and consist mainly of one type of repeat, e.g., *Arabidopsis thaliana*, that displays a strong preference for a unique subset of centromeric CEN180 satDNA repeats [70]. On the contrary, some organisms show some diversity in CenH3-associated sequences, encompassing different types of repetitive DNA, as presented here for *C. gigas*, and reported also for *Triticum aestivum* [71], *Beta vulgaris* [38], *Saccharum spontaneum* [39], *Oryza sativa* [72] or *Drosophila melanogaster* [73]. 

The information regarding Cg-CenH3 and H3K9me3-associated DNA sequences put forward in this work disclosed general features and several constituents of the poorly characterized centromeric chromatin and heterochromatin of the Pacific oyster. The general paucity and limited localization of heterochromatin constituted predominantly of mobile elements, and the indicated lack of uniformity in repetitive DNA sequences constituting the centromeres also found dispersed throughout the genome, are features promoting *C. gigas* as an exceptional model in studying centromere as well as heterochromatin genomics. We hope that further research, employing third-generation sequencing producing long reads, encompassing centromeric and heterochromatic areas, will provide details of longitudinal arrangements of DNA sequences in these parts of the genome of this economically and ecologically important organism.

## Figures and Tables

**Table 1:** Annotation summary of the most prominent repetitive sequences of *C. gigas* accessed by RepeatExplorer clustering.

Repeat	% of the Genome
**satellite**	**2.56**
Cg170 satDNA	1.76
**mobile elements**	**2.07**
Ty3 gypsy	0.09
DIRS	0.04
LINE	0.11
Penelope	0.04
**45S rDNA**	**0.40**
**mitochondria**	**0.02**
**Unclassified**	**15.64**

## Supplementary Materials

The following are available online at https://www.mdpi.com/2073-4425/11/6/695/s1, Figure S1: The protein sequence alignment of *C. gigas* H3 and Cg-CenH3 and Cg-CenH3 antibody testing by western blot, Figure S2: Annotation of Cg170 repeats on pseudochromosomes of *C. gigas*., Figure S3: IF colocalization of Cg-CenH3 and H3K9me3 on male gonadal cells of *C. gigas.*, Table S1: Primers and parameters used for PCR reaction for probe labelling, Table S2: Contigs enriched in the Cg-CenH3 ChIP samples and their classification by Repbase and RepeatExplorer, Table S3: Helitron elements whose central parts are constituted of Cg170 tandem repeats, Table S4: Contigs enriched in the H3K9me3 ChIP sample and their classification by Repbase and RepeatExplorer.

## References

[1] Vaughn C.C., Hoellein T.J. (2018). Bivalve Impacts in Freshwater and Marine Ecosystems. Annu. Rev. Ecol. Evol. Syst..

[2] Zhang G., Fang X., Guo X., Li L., Luo R., Xu F., Yang P., Zhang L., Wang X., Qi H. (2012). The Oyster Genome Reveals Stress Adaptation and Complexity of Shell Formation. Nature.

[3] Plohl M., Meštrović N., Mravinac B. (2014). Centromere Identity from the DNA Point of View. Chromosoma.

[4] McKinley K.L., Cheeseman I.M. (2016). The Molecular Basis for Centromere Identity and Function. Nat. Rev. Mol. Cell Biol..

[5] Malik H.S., Henikoff S. (2009). Major Evolutionary Transitions in Centromere Complexity. Cell.

[6] Jiang J., Birchler J.A., Parrott W.A., Dawe R.K. (2003). A Molecular View of Plant Centromeres. Trends Plant Sci..

[7] Šatović E., Vojvoda Zeljko T., Plohl M. (2018). Characteristics and Evolution of Satellite DNA Sequences in Bivalve Mollusks. Eur. Zool. J..

[8] Clabby C., Goswami U., Flavin F., Wilkins N.P., Houghton J.A., Powell R. (1996). Cloning, Characterization and Chromosomal Location of a Satellite DNA from the Pacific Oyster, *Crassostrea gigas*. Gene.

[9] Wang Y., Xu Z., Guo X. (2001). A Centromeric Satellite Sequence in the Pacific Oyster (*Crassostrea gigas* Thunberg) Identified by Fluorescence In Situ Hybridization. Mar. Biotechnol..

[10] López-Flores I., de la Herrán R., Garrido-Ramos M.A., Boudry P., Ruiz-Rejón C., Ruiz-Rejón M. (2004). The Molecular Phylogeny of Oysters Based on a Satellite DNA Related to Transposons. Gene.

[11] Luchetti A., Šatović E., Mantovani B., Plohl M. (2016). RUDI, a Short Interspersed Element of the V-SINE Superfamily Widespread in Molluscan Genomes. Mol. Genet. Genomics.

[12] Thomas-Bulle C., Piednoël M., Donnart T., Filée J., Jollivet D., Bonnivard É. (2018). Mollusc Genomes Reveal Variability in Patterns of LTR-Retrotransposons Dynamics. BMC Genomics.

[13] Šatović E., Luchetti A., Pasantes J.J., García-Souto D., Cedilak A., Mantovani B., Plohl M. (2019). Terminal-Repeat Retrotransposons in Miniature (TRIMs) in Bivalves. Sci. Rep..

[14] Gaffney P.M., Pierce J.C., Mackinley A.G., Titchen D.A., Glenn W.K. (2003). Pearl, a Novel Family of Putative Transposable Elements in Bivalve Mollusks. J. Mol. Evol..

[15] Kourtidis A., Drosopoulou E., Pantzartzi C.N., Chintiroglou C.C., Scouras Z.G. (2006). Three New Satellite Sequences and a Mobile Element Found inside HSP70 Introns of the Mediterranean Mussel (*Mytilus galloprovincialis*). Genome.

[16] Šatović E., Plohl M. (2013). Tandem Repeat-Containing MITE Elements in the Clam *Donax trunculus*. Genome Biol. Evol..

[17] Šatović E., Plohl M. (2017). Two New Miniature Inverted-Repeat Transposable Elements in the Genome of the Clam *Donax trunculus*. Genetica.

[18] Meštrović N., Mravinac B., Pavlek M., Vojvoda-Zeljko T., Šatović E., Plohl M. (2015). Structural and Functional Liaisons between Transposable Elements and Satellite DNAs. Chromosom. Res..

[19] Dias G.B., Heringer P., Svartman M., Kuhn G.C.S.S. (2015). Helitrons Shaping the Genomic Architecture of *Drosophila*: Enrichment of DINE-TR1 in α- and β-Heterochromatin, Satellite DNA Emergence, and piRNA Expression. Chromosom. Res..

[20] Luchetti A. (2015). TerMITEs: Miniature Inverted-Repeat Transposable Elements (MITEs) in the Termite Genome (Blattodea: Termitoidae). Mol. Genet. Genomics.

[21] Plohl M., Petrović V., Luchetti A., Ricci A., Šatović E., Passamonti M., Mantovani B. (2010). Long-Term Conservation vs High Sequence Divergence: The Case of an Extraordinarily Old Satellite DNA in Bivalve Mollusks. Heredity (Edinb).

[22] Šatović E., Vojvoda Zeljko T., Luchetti A., Mantovani B., Plohl M. (2016). Adjacent Sequences Disclose Potential for Intra-Genomic Dispersal of Satellite DNA Repeats and Suggest a Complex Network with Transposable Elements. BMC Genomics.

[23] Thomas J., Pritham E.J. (2015). Helitrons, the Eukaryotic Rolling-Circle Transposable Elements. Microbiol. Spectr..

[24] Nicetto D., Zaret K.S. (2019). Role of H3K9me3 Heterochromatin in Cell Identity Establishment and Maintenance. Curr. Opin. Genet. Dev..

[25] Sullivan B.A., Karpen G.H. (2004). Centromeric Chromatin Exhibits a Histone Modification Pattern That Is Distinct from Both Euchromatin and Heterochromatin. Nat. Struct. Mol. Biol..

[26] Havenhand J.N., Li X.X. (1997). Karyotype, Nucleolus Organiser Regions and Constitutive Heterochromatin in *Ostrea angasi* (Molluscae: Bivalvia): Evidence of Taxonomic Relationships within the Ostreidae. Mar. Biol..

[27] Cross I., Díaz E., Sánchez I., Rebordinos L. (2005). Molecular and Cytogenetic Characterization of *Crassostrea angulata* Chromosomes. Aquaculture.

[28] Petrović V., Pérez-García C., Pasantes J.J., Šatović E., Prats E., Plohl M. (2009). A GC-Rich Satellite DNA and Karyology of the Bivalve Mollusk *Donax trunculus*: A Dominance of GC-Rich Heterochromatin. Cytogenet. Genome Res..

[29] Petkevičiūtė R., Stunžėnas V., Stanevičiūtė G. (2018). Comments on Species Divergence in the Genus *Sphaerium* (Bivalvia) and Phylogenetic Affinities of *Sphaerium nucleus* and *S. corneum* var. *mamillanum* Based on Karyotypes and Sequences of 16S and ITS1 RDNA. PLoS ONE.

[30] Bouilly K., Chaves R., Leitao A., Benabdelmouna A., Guedes-Pinto H. (2008). Chromosomal Organization of Simple Sequence Repeats in Chromosome Patterns. J. Genet..

[31] Sedlazeck F.J., Lee H., Darby C.A., Schatz M.C. (2018). Piercing the Dark Matter: Bioinformatics of Long-Range Sequencing and Mapping. Nat. Rev. Genet..

[32] Gagnaire P.-A., Lamy J.-B., Cornette F., Heurtebise S., Dégremont L., Flahauw E., Boudry P., Bierne N., Lapègue S. (2018). Analysis of Genome-Wide Differentiation between Native and Introduced Populations of the Cupped Oysters *Crassostrea gigas* and *Crassostrea angulata*. Genome Biol. Evol..

[33] Bouilly K., Chaves R., Fernandes M., Guedes-Pinto H. (2010). Histone H3 Gene in the Pacific Oyster, *Crassostrea gigas* Thunberg, 1793: Molecular and Cytogenetic Characterisations. Comp. Cytogenet..

[34] Liu J., Li Q., Kong L., Yu H., Zheng X. (2011). Identifying the True Oysters (Bivalvia: Ostreidae) with Mitochondrial Phylogeny and Distance-Based DNA Barcoding. Mol. Ecol. Resour..

[35] Folmer O., Black M., Hoeh W., Lutz R., Vrijenhoek R. (1994). DNA Primers for Amplification of Mitochondrial Cytochrome c Oxidase Subunit I from Diverse Metazoan Invertebrates. Mol. Mar. Biol. Biotechnol..

[36] Novák P., Robledillo L.Á., Koblížková A., Vrbová I., Neumann P., Macas J. (2017). TAREAN: A Computational Tool for Identification and Characterization of Satellite DNA from Unassembled Short Reads. Nucleic Acids Res..

[37] Novák P., Neumann P., Pech J., Steinhaisl J., Macas J. (2013). RepeatExplorer: A Galaxy-Based Web Server for Genome-Wide Characterization of Eukaryotic Repetitive Elements from next-Generation Sequence Reads. Bioinformatics.

[38] Kowar T., Zakrzewski F., Macas J., Kobližková A., Viehoever P., Weisshaar B., Schmidt T. (2016). Repeat Composition of CenH3-Chromatin and H3K9me2-marked Heterochromatin in Sugar Beet (*Beta vulgaris*). BMC Plant Biol..

[39] Zhang W., Zuo S., Li Z., Meng Z., Han J., Song J., Pan Y.B., Wang K. (2017). Isolation and Characterization of Centromeric Repetitive DNA Sequences in *Saccharum spontaneum*. Sci. Rep..

[40] Martínez-Expósito M.J., Pasantes J.J., Méndez J. (1994). NOR Activity in Larval and Juvenile Mussels (*Mytilus galloprovincialis* Lmk.). J. Exp. Mar. Biol. Ecol..

[41] Franco A., Heude Berthelin C., Goux D., Sourdaine P., Mathieu M. (2008). Fine Structure of the Early Stages of Spermatogenesis in the Pacific Oyster, *Crassostrea gigas* (Mollusca, Bivalvia). Tissue Cell.

[42] Pérez-García C., Morán P., Pasantes J.J. (2011). Cytogenetic Characterization of the Invasive Mussel Species *Xenostrobus securis* Lmk. (Bivalvia: Mytilidae). Genome.

[43] Murgarella M., Puiu D., Novoa B., Figueras A., Posada D., Canchaya C. (2016). A First Insight into the Genome of the Filter-Feeder Mussel *Mytilus galloprovincialis*. PLoS ONE.

[44] Silva B.S.M.L., Heringer P., Guilherme B.D., Svartman M., Kuhn G.C.S. (2019). De Novo Identification of Satellite DNAs in the Sequenced Genomes of *Drosophila virilis* and *D. americana* Using the RepeatExplorer and TAREAN Pipeline. PLoS ONE.

[45] Abdurashitov M.A., Gonchar D.A., Chernukhin V.A., Tomilov V.N., Tomilova J.E., Schostak N.G., Zatsepina O.G., Zelentsova E.S., Evgen′ev M.B., Degtyarev S.K. (2013). Medium-Sized Tandem Repeats Represent an Abundant Component of the *Drosophila virilis* Genome. BMC Genomics.

[46] Zelentsova E.S., Vashakidze R.P., Krayev A.S., Evgen′ev M.B. (1986). Dispersed Repeats in *Drosophila virilis*: Elements Mobilized by Interspecific Hybridization. Chromosoma.

[47] Xiong W., Dooner H.K., Du C. (2016). Rolling-Circle Amplification of Centromeric Helitrons in Plant Genomes. Plant J..

[48] Gong Z., Wu Y., Koblížková A., Torres G.A., Wang K., Iovene M., Neumann P., Zhang W., Novák P., Robin Buell C. (2012). Repeatless and Repeat-Based Centromeres in Potato: Implications for Centromere Evolution. Plant Cell.

[49] Mehra M., Gangwar I., Shankar R. (2015). A Deluge of Complex Repeats: The Solanum Genome. PLoS ONE.

[50] Long E.O., Dawid I.B. (1980). Repeated Genes in Eukaryotes. Ann. Rev. Biochem..

[51] Stupar R.M., Song J., Tek A.L., Cheng Z., Dong F., Jiang J. (2002). Highly Condensed Potato Pericentromeric Heterochromatin Contains rDNA-Related Tandem Repeats. Genetics.

[52] Lim K.Y., Matyášek R., Lichtenstein C.P., Leitch A.R. (2000). Molecular Cytogenetic Analyses and Phylogenetic Studies in the *Nicotiana* Section Tomentosae. Chromosoma.

[53] Jo S.H., Koo D.H., Kim J.F., Hur C.G., Lee S., Yang T.J., Kwon S.Y., Choi D. (2009). Evolution of Ribosomal DNA-Derived Satellite Repeat in Tomato Genome. BMC Plant Biol..

[54] Almeida C., Fonsêca A., dos Santos K.G.B., Mosiolek M., Pedrosa-Harand A. (2012). Contrasting Evolution of a Satellite DNA and Its Ancestral IGS rDNA in Phaseolus (Fabaceae). Genome.

[55] Pérez-García C., Morán P., Pasantes J.J. (2014). Karyotypic Diversification in *Mytilus* Mussels (Bivalvia: Mytilidae) Inferred from Chromosomal Mapping of rRNA and Histone Gene Clusters. BMC Genet..

[56] García-Souto D., Pérez-García C., Morán P., Pasantes J.J. (2015). Divergent Evolutionary Behavior of H3 Histone Gene and rDNA Clusters in Venerid Clams. Mol. Cytogenet..

[57] García-Souto D., Ríos G., Pasantes J.J. (2017). Karyotype Differentiation in Tellin Shells (Bivalvia: Tellinidae). BMC Genet..

[58] García-Souto D., Qarkaxhija V., Pasantes J.J. (2017). Resolving the Taxonomic Status of *Chamelea gallina* and *C. striatula* (Veneridae, Bivalvia): A Combined Molecular Cytogenetic and Phylogenetic Approach. BioMed Res. Int..

[59] García-Souto D., Pasantes J.J. (2018). Cytogenetics in *Arctica islandica* (Bivalvia, Arctidae): The Longest Lived Non-Colonial Metazoan. Genes (Basel).

[60] Layat E., Sáez-Vásquez J., Tourmente S. (2012). Regulation of Pol I-Transcribed 45S rDNA and Pol III-Transcribed 5S rDNA in *Arabidopsis*. Plant Cell Physiol..

[61] Robicheau B.M., Susko E., Harrigan A.M., Snyder M. (2017). Ribosomal RNA Genes Contribute to the Formation of Pseudogenes and Junk DNA in the Human Genome. Genome Biol. Evol..

[62] Yang X., Zhao H., Zhang T., Zeng Z., Zhang P., Zhu B., Han Y., Braz G.T., Casler M.D., Schmutz J. (2018). Amplification and Adaptation of Centromeric Repeats in Polyploid Switchgrass Species. New Phytol..

[63] Scherthan H., Weich S., Schwegler H., Heyting C., Härle M., Cremer T. (1996). Centromere and Telomere Movements during Early Meiotic Prophase of Mouse and Man are Associated with the Onset of Chromosome Pairing. J. Cell Biol..

[64] Colaco S., Modi D. (2018). Genetics of the Human Y Chromosome and Its Association with Male Infertility. Reprod. Biol. Endocrinol..

[65] Matsunaga S., Takata H., Morimoto A., Hayashihara K., Higashi T., Akatsuchi K., Mizusawa E., Yamakawa M., Ashida M., Matsunaga T.M. (2012). RBMX: A Regulator for Maintenance and Centromeric Protection of Sister Chromatid Cohesion. Cell Rep..

[66] Cho Y., Ideue T., Nagayama M., Araki N., Tani T. (2018). RBMX Is a Component of the Centromere Noncoding RNP Complex Involved in Cohesion Regulation. Genes Cells.

[67] Wiland E., Fraczek M., Olszewska M., Kurpisz M. (2016). Topology of Chromosome Centromeres in Human Sperm Nuclei with High Levels of DNA Damage. Sci. Rep..

[68] Gosálvez J., Kjelland M.E., López-Fernández C. (2010). Sperm DNA in Grasshoppers: Structural Features and Fertility Implications. J. Orthoptera Res..

[69] Champroux A., Damon-Soubeyrand C., Goubely C., Bravard S., Henry-Berger J., Guiton R., Saez F., Drevet J., Kocer A. (2018). Nuclear Integrity but Not Topology of Mouse Sperm Chromosome Is Affected by Oxidative DNA Damage. Genes.

[70] Maheshwari S., Ishii T., Brown C.T., Houben A., Comai L. (2017). Centromere Location in *Arabidopsis* Is Unaltered by Extreme Divergence in CENH3 Protein Sequence. Genome Res..

[71] Su H., Liu Y., Liu C., Shi Q., Huang Y., Han F. (2019). Centromere Satellite Repeats Have Undergone Rapid Changes in Polyploid Wheat Subgenomes. Plant Cell.

[72] Yan H., Ito H., Nobuta K., Ouyang S., Jin W., Tian S., Lu C., Venu R.C., Wang G.L., Green P.J. (2006). Genomic and Genetic Characterization of Rice Cen3 Reveals Extensive Transcription and Evolutionary Implications of a Complex Centromere. Plant Cell.

[73] Chang C.-H., Chavan A., Palladino J., Wei X., Martins N.M.C., Santinello B., Chen C.-C., Erceg J., Beliveau B.J., Wu C.-T. (2019). Islands of Retroelements Are Major Components of *Drosophila* Centromeres. PLoS Biol..

